# An Efficient Photodynamic Therapy Treatment for Human Pancreatic Adenocarcinoma

**DOI:** 10.3390/jcm9010192

**Published:** 2020-01-10

**Authors:** Alexandre Quilbe, Olivier Moralès, Martha Baydoun, Abhishek Kumar, Rami Mustapha, Takashi Murakami, Bertrand Leroux, Clémentine de Schutter, Elise Thecua, Laurine Ziane, Ludovic Colombeau, Céline Frochot, Serge Mordon, Nadira Delhem

**Affiliations:** 1 CNRS, UMR8161, Institut de Biologie de Lille, Université de Lille, Institut Pasteur de Lille, F-59021 Lille, France; alexandre.quilbe@ibl.cnrs.fr (A.Q.); olivier.morales@ibl.cnrs.fr (O.M.); martha.baydoun@ibl.cnrs.fr (M.B.); abhishek.kumar@ibl.cnrs.fr (A.K.); bertrand.leroux@inserm.fr (B.L.); clementine.de-schutter@ibl.cnrs.fr (C.d.S.); 2 Univ.Lille, Inserm, CHU Lille, U1189, ONCO-THAI- Image Assisted Laser Therapy for Oncology, PhysicoPDT team or Immuno-PDT team, F-59000 Lille, France; Elise.thecua@inserm.fr (E.T.); laurine.ziane@inserm.fr (L.Z.); 3 Department of Cancer Studies & Pharmaceutical Sciences New Hunt’s House, School of Life Sciences and Medicine, Guy’s Campus, King’s College London, London SE1 1UL, UK; 4 Faculty of Medicine, Saitama Medical University 38 Moro-Hongo, Moroyama, Ituma, Saitama 350-0495, Japan; t_murakami@saitama-med.ac.jp; 5 LGRGP, UMR-CNRS 7274, University of Lorraine, F-54000 Nancy, France; ludovic.colombeau@univ-lorraine.fr (L.C.); celine.frochot@univ-lorraine.fr (C.F.)

**Keywords:** photodynamic therapy, folate-coupled photosensitizer, pancreatic cancer, immuno-adjuvant

## Abstract

To date, pancreatic adenocarcinoma (ADKP) is a devastating disease for which the incidence rate is close to the mortality rate. The survival rate has evolved only 2–5% in 45 years, highlighting the failure of current therapies. Otherwise, the use of photodynamic therapy (PDT), based on the use of an adapted photosensitizer (PS) has already proved its worth and has prompted a growing interest in the field of oncology. We have developed a new photosensitizer (PS-FOL/PS2), protected by a recently published patent (WO2019 016397-A1, 24 January 2019). This photosensitizer is associated with an addressing molecule (folic acid) targeting the folate receptor 1 (FOLR1) with a high affinity. Folate binds to FOLR1, in a specific way, expressed in 100% of ADKP or over-expressed in 30% of cases. The first objective of this study is to evaluate the effectiveness of this PS2-PDT in four ADKP cell lines: Capan-1, Capan-2, MiapaCa-2, and Panc-1. For this purpose, we first evaluated the gene and protein expression of FOLR1 on four ADKP cell lines. Subsequently, we evaluated PS2’s efficacy in our cell lines and we assessed the impact of PDT on the secretome of cancer cells and its impact on the immune system. Finally, we evaluate the PDT efficacy on a humanized SCID mouse model of pancreatic cancer. In a very interesting way, we observed a significant increase in the proliferation of activated-human PBMC when cultured with conditioned media of ADKP cancer cells subjected to PDT. Furthermore, to evaluate in vivo the impact of this new PS, we analyzed the tumor growth in a humanized SCID mice model of pancreatic cancer. Four conditions were tested: Untreated, mice (nontreated), mice with PS (PS2), mice subjected to illumination (Light only), and mice subjected to illumination in the presence of PS (PDT). We noticed that the mice subjected to PDT presented a strong decrease in the growth of the tumor over time after illumination. Our investigations have not only suggested that PS2-PDT is an effective therapy in the treatment of PDAC but also that it activates the immune system and could be considered as a real adjuvant for anti-cancer vaccination. Thus, this new study provides new treatment options for patients in a therapeutic impasse and will provide a new arsenal in the fight against PDAC.

## 1. Introduction

To date, pancreatic adenocarcinoma (PDAC) is a devastating disease for which the incidence rate is close to the mortality rate. The survival rate has only increased by 2% to 5% in 45 years, highlighting the failure of current therapies. Pancreatic cancer is classified into two main types: Those that form in the exocrine gland and those that form in the endocrine gland. We are interested particularly in the exocrine pancreatic cancer known as pancreatic ductal adenocarcinoma (PDAC) presenting more than 90% of all forms of cancer [1,2,3,4]. In France, the estimated incidence for 2017 was 14,200 cases with a male/female ratio of 1.48. The peak incidence is between 70 and 80 years, whereas the risk of developing pancreatic cancer is low until age 50 (5% of cases). It is estimated that it will become the second leading cause of cancer deaths in France by 2030 [5,6].

Since this tumor progresses rapidly with few specific symptoms, PDAC is often diagnosed at an advanced stage and the surgery procedures are only performed in only 20% to 30% of patients [7]. It mainly concerns small tumors confined to the pancreas (less than 2 to 2.5 cm in diameter), without metastasis or invasion of the vessels and arteries. The five-year survival rate after surgery is 15% to 20%. An adjuvant chemotherapy for three months can be proposed hence allowing a delay in recurrence and increase in the overall survival (17 vs. 23 months) [5]. Radiation therapy is also used as a neo-adjuvant before surgery to facilitate it and destroy locally disseminated cancer cells. Chemotherapy is offered when surgery is not possible. Statistically more than 60% of the patients are diagnosed at the metastatic stage, 25% at a locally advanced stage without metastasis, and 15% at a surgically respectable stage or after neoadjuvant radio-chemotherapy [8,9].

However, the incidence has doubled in the past 10 years and no therapeutic innovation has significantly improved the prognosis of this disease. There is an urgent need for alternative therapeutic innovation in order to improve the prognosis of this cancer.

Photodynamic treatment (PDT) for pancreatic carcinoma have shown great potential in reducing tumor volume and prolongation of patient’s survival [7]. In fact, the addition of PDT to surgery is believed to improve the local control by eradicating the remaining metastatic cancer cells [10]. In general, the considerable improvement of PDT compared to standard treatment protocols for cancer are higher selectivity and lower toxicity. Several PDT strategies have been tested in human PDAC using mTHPC [11] and Photofrin [12]. However, due to the lack of specificity for tumor cells, PDT in PDAC caused serious side effects, such as duodenal perforation and liver lesions. In this context, it seems necessary to develop a new PS more selective and specific of PDAC cells.

In order to improve this selectivity, we have patented a photosentizer coupled with folate receptor (FR) (WO/2019/016397). Four isoforms of FR (α, β, γ, and δ) have been described but FR-α was essentially found on cancers of the ovary, breast, head and neck, endometrium, lung, bladder, colon, kidney and also pancreas [13,14,15]. It has previously been shown that FRα expression intensity in 140 PDACs patients was low, intermediate, and high in 22 (16%), 73 (52%), and 45 (32%), respectively. Thus, FRα in PDACs may represent a promising target for novel treatments [13]. In this study, we are interested in validating both in vitro and in vivo the use of our new PS for the treatment of pancreatic cancer. Furthermore, we investigate the potential immunostimulating effect of PDT using this new-patented PS on human leukocytes.

## 2. Materials and Methods

### 2.1. Cell Culture

PDAC cell lines Panc-1, Capan-1, and Capan-2 were kindly provided by the team of Dr. Isabelle Van Seuningen and Dr. Nicolas Jonckheere (Centre de recherche Jean-Pierre Aubert, Lille, France). MiaPaCa-2-Luc cell line was ordered from the JRCB cell bank. The ovarian tumor line Ovcar-3 was ordered from the American Type Culture Collection (ATCC). Ovcar-3, Capan-1, and Capan-2 cells were cultured in a Roswell Park Memorial Institute medium (RPMI 1640, Gibco, France) supplemented respectively with 10%, 10%, and 20% (v/v) of heat decomplemented fetal calf serum (Eurobio, Les Ulis, France). Panc-1 and MiaPaCa-2 cells were cultured in Dulbecco’s modified Eagle’s medium supplemented with 10% fetal calf serum. All the media are supplemented with 100 units/mL penicillin, 100 µg/mL streptomycin, and 2 mM glutamine (Life Technologies, Carlsbad, CA, USA). Cells were maintained at 37 °C, 5% CO_2_, and 95% humidity.

### 2.2. Photodynamic Therapy Protocol

One million cells and five hundred cells were respectively seeded in a flask 25 cm^2^ and 96-well plates. 24 h post-treatment, the medium is replaced by a medium containing the PS2 (1 mg/mL). After 24 h, the medium containing the PS2 is changed and replaced by the normal medium after two washes with PBS-/- (Life Technologies; Carlsbad, CA, USA). The cells were then subjected to 673 nm laser for 1 h. 24 h later the supernatant is recovered, centrifuged, and then frozen at −20 °C. Four groups of cells were used: Untreated tumor cells (Non treated), treated with PS without illumination (PS2), illuminated cells only (+light only), and cells subjected to PDT (PDT). PS2 was synthesized and provided by the team of Dr. Celine Frochot (Supplementary Figure S1). In vitro and in vivo illumination was performed as previously described with a specific device developed by OncoThAI research team [16]. For in vitro study, a homogenous illumination during 1 h at 1 mW/cm^2^ (3.6 J/cm^2^) was performed and for in vivo treatment, an extracorporeal fractionated illumination of 2 h 30 in total (1 min of illumination followed by 2 min of rest, repeated 45 times) at 11 mW/cm^2^ (29.7 J/cm^2^) was performed.

### 2.3. RNA Extraction

Total RNA extraction of the cancer cell lines was performed using the RNeasy mini kit (QIAGEN, Courtaboeuf, France), as described by the supplier. Briefly, and after lysing the sample, ethanol was added to the lysate in order to grant optimal binding conditions. The lysate was then efficiently washed and loaded onto the RNeasy silica membrane for RNA binding, all contaminants were hence discharged. Pure, concentrated RNA was eluted in water and stored at −80 °C.

### 2.4. Retro-Transcription (RT) and Quantitative PCR

The SuperscriptTM II Transcriptase Reverse Kit is used for RT (Invitrogen, Carlsbad, CA, USA) GB. Reverse transcription was performed using 1 µg of total RNA. The RT-PCR reactions were performed, for selected genes (Table 1), according to the manufacturer’s instructions using 2× MESA GREEN qPCR MasterMix Plus for SYBR 258 Assay (Eurogentech, Liège, Belgique) 96-well qPCR plate (Sarstedt, Nümbrecht, Germany), optical seal (Dutcher, Brumath, France), and the Mx3005PTM sequence detection system (Agilent technologies, Santa Clara, CA, USA). In each reaction, 10 ng of reverse transcripted RNA (based on initial RNA concentration) was used. All primers were used at 400 nM in a 20 µL reaction. Quantitative analysis was made based on the cycle threshold (Ct) value for each well and calculated using the MxPro software (Agilent technologies, Santa Clara, CA, USA). The results are normalized by three housekeeping (HKG) genes: 18S, GAPDH, and HPRT (Table 1), and data are represented as fold differences by the 2^−ΔΔCt^ method, where ΔCt = Ct target gene—Ct HKG.

### 2.5. Viability Test

The cells were cultured, in 96-well Costar plates (Corning, Corning, NY, USA) (30,000 Cells/mL) in triplicate for each condition, (Non treated, light only, PS2, PDT). 24 h post PDT 100 µL/well of Celltiter-Glo mix (Promega, Charbonnières-les-Bains, France) has been added, at room temperature for 10 min and protected from light. Then, the luminescence reading was taken with the Luminometer centro LB960 (Berthold Technologies, Oak Ridge, TN, USA)

### 2.6. Immunofluorescence

10^4^ cells were cultured on sterile lamellae 10 mm in diameter in 12-well plates (Dutscher, France). 24 h after culture, the medium was renewed with sterile medium containing PS2 at 1 µg/mL for 24 h. Negative controls, not containing PS2, were carried out. The cells were washed three times with PBS-/- and then fixed with a solution containing 4% paraformaldehyde (PAF) (Santa Cruz Biotechnology, Dallas, TX, USA). The cells incubated 10 min with 50 ng/mL DAPI (Sigma-Aldrich, Saint-Louis, MO, USA) to obtain a nuclear dye and then washed with PBS-/-. The slides containing the cells were then mounted on slides using Mowiol (Sigma-Aldrich, Saint-Louis, MO, USA) and stored at 4 °C until analysis. The analysis was carried using a confocal microscope LSM 880 (Carl Zeiss, Oberkochen, Germany) with a magnification of 63×. The images were acquired with an excitation wavelength of 405 nm and an emission wavelength between 630 and 700 nm powered by Zen Lite 2.3 (Zeiss, Oberkochen, Germany).

### 2.7. Flow Cytometry

FOLR1 receptor expression was analyzed by cytometry on all four PDAC cell lines and the OVCAR3 cell line as a positive control. We used an anti-FOLR1-PE (BioLegend, San Diego, CA, USA) and its IgG2a-PE isotype control (Miltenyi Biotec, Bergisch Gladbach, Germany) to determine the expression of the FOLR1 receptor. 10^4^ cells were taken up in a volume of 200 μL of PBS-/- and the fragment crystallizable receptors (FCR) were blocked with the FCR blocking reagent (Miltenyi Biotec, Bergisch Gladbach, Germany) for 15 min at 4 °C. They were then incubated for 15 min at 4 °C and in the dark with 2 μL of each antibody. The labelled cells were filled up with 300 μL of PBS-/- and the fluorescence was analyzed by flow cytometry at the BD FACS Canto IITM (Becton Dickinson, Franklin Lakes, NJ, USA). The cytometry results were then analyzed with the Flow Jo software (Becton Dickinson, Franklin Lakes, NJ, USA). The results are expressed as an MFI ratio (RFI).

### 2.8. Isolation of Human Healthy Donor PBMCs

Human blood samples were collected from healthy adult donors after obtaining informed consent in accordance with the approval of the Institutional Review Board at the Biology Institute of Lille (DC-2013-1919). Peripheral blood mononuclear cells (PBMC) were isolated from peripheral blood samples by density gradient centrifugation using a lymphocytes separation medium (Eurobio, Les Ullis, France) and leucosep 50 mL tubes (Greiner Bio One, Courtaboeuf, France).

### 2.9. Proliferation Test

10^5^ PBMCs were cultivated in a ML10 medium made with RPMI 1640 medium supplemented with sodium pyruvate (1 mM), nonessential amino acids MEM 1×, HEPES (25 mM), 2-mercaptoethanol (50 μM), gentamicin (10 μg/mL) (Thermo Fisher Scientific, Waltham, MA, USA), and 10% SVF (GIBCO, Invitrogen). The cells were activated with plated anti-CD3 (1 µg/mL (Miltenyi, Bergisch Gladbach, Germany)) and anti-CD28 (100 ng/mL, (Clinisciences, MontRouge, France). Cancer cell lines supernatant (treated or not) also called conditioned media (50 µL/well) was added to PBMC in 96 round-bottomed plates for 48, 72, 96, 120 h. Proliferation was measured after incorporation of (3H) thymidine (1 µCi/well) (PerkinElmer, Courtaboeuf, France) for 18 h before the end of the culture. Radioactivity was determined using a β-counter (1450 Trilux, Wallac, Finland).

### 2.10. ELISA

The cytokine detections were carried out on the supernatants of cancer cell lines treated or not treated with PDT: Supernatant of untreated cells, illuminated cells, in contact with PS, cells subjected to PDT. Supernatants of all cell cultures were harvested and kept at −80 °C until their use for cytokine assays. Cytokine secretions of interleukin (IL)-6, transforming growth factor (TGF)-β1, IL2, and interferon (IFN)-γ were determined by the Sandwich ELISA (Enzyme-Linked Immunosorbent Assay) method. Briefly, purified primary antibodies were coated overnight at 4 °C in flat bottom 96-well plates (NUNC, Danemark) before incubation with samples. The corresponding biotinylated antibodies were added for protein detection, after several steps of specific sites blocking, samples deposit (overnight at 4 °C), and adequate washings (PBS-Tween 0.05%). The reaction was amplified with Streptavidine-peroxydase (Interchim, Montluçon, France). Cytokines concentration was finally highlighted with the addition of OPD (10 mg/mL, Sigma-Aldrich, St Louis, MO, USA). After color development, the plates were read in a Multiskan spectrophotometer at 492 nm powered by Ascent™ Software v2.06 (Multiskan RC Thermo Labsystems, Thermo Fisher Scientific, Waltham, MA, USA). The purified and biotinylated antibodies used were the following: Mouse anti-human IL2, rat anti-human IL6, rat anti-human TGFβ1, and mouse anti-IFN-γ (all from BD PharmingenTM, San Jose, CA, USA). Results were expressed in pg/mL as the mean of triplicates wells after subtracting background values.

### 2.11. Humanized SCID Mice Model

All procedures were approved by the local Ethical Committee of the IPL performed with required permission of the regional governing ethical board (approval number CEEA 152010). Anesthetized SCID mice were subcutaneously xenotransplanted with an IVIS LUMINA XR reader (Caliper Life Sciences, Hopkinton, MA, USA). Mice were divided in four groups which received: Mothing, light only, PS2 only, PS2, and Light (PDT). For PS2 only and PDT condition 100 μL of a solution at 1 mg/mL of PS2 was injected intraperitoneally. Images were then analyzed under the Living Image 4.1 software (Caliper Life Sciences, Hopkinton, MA, USA) and results were obtained after spectral unmixing according to the manufacturer’s instructions. Measurements of tumor bioluminescence were acquired at different times: Day 1, 3, 5, 7, 9, 12 to monitor tumor growth, after i.p. injection of 100 µL of D-luciferin (30 mg/mL, Perkin Elmer, Waltham, MA, USA) into each mouse before analysis.

### 2.12. Statistical Analysis

Data were analyzed using the statistical package GraphPad for windows 3.0.1. All quoted *p*-values are two-sided, with *p* ≤ 0.05 (*), *p* ≤ 0.001 (**), *p* ≤ 0.0001 (***), and *p* ≤ 0.00001 (****) being considered statistically significant for the first and highly significant for the others.

## 3. Results

### 3.1. FOLR1 Gene and Protein Expression by PDAC Cell Lines

**Figure 1:**
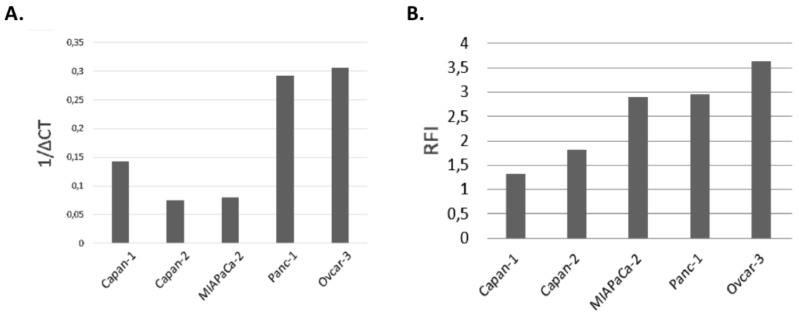
Transcriptomic and protein expression of folate receptor 1 (FOLR1) in pancreatic ductal adenocarcinoma (PDAC) cell lines and one ovarian cancer (positive control). (**A**) Expression of FOLR1 by RT-qPCR. (**B**) Membrane protein expression of FOLR1 by flow cytometry (RFI).

We evaluated by RT-qPCR the expression of FOLR1 transcripts in four cancer cell lines: MiaPaCa-2, Panc-1, Capan-1, and Capan-2. (Figure 1A). FOLR1 transcripts is expressed in the four different cancer cell lines (Figure 1A). Cytometry analysis confirmed the protein expression of FOLR1 by the four cancer cell lines. Panc-1 and MiaPaCa-2 express more FOLR1 protein (Figure 1B).

### 3.2. PS2 Localization in PDAC Cell Lines

**Figure 2:**
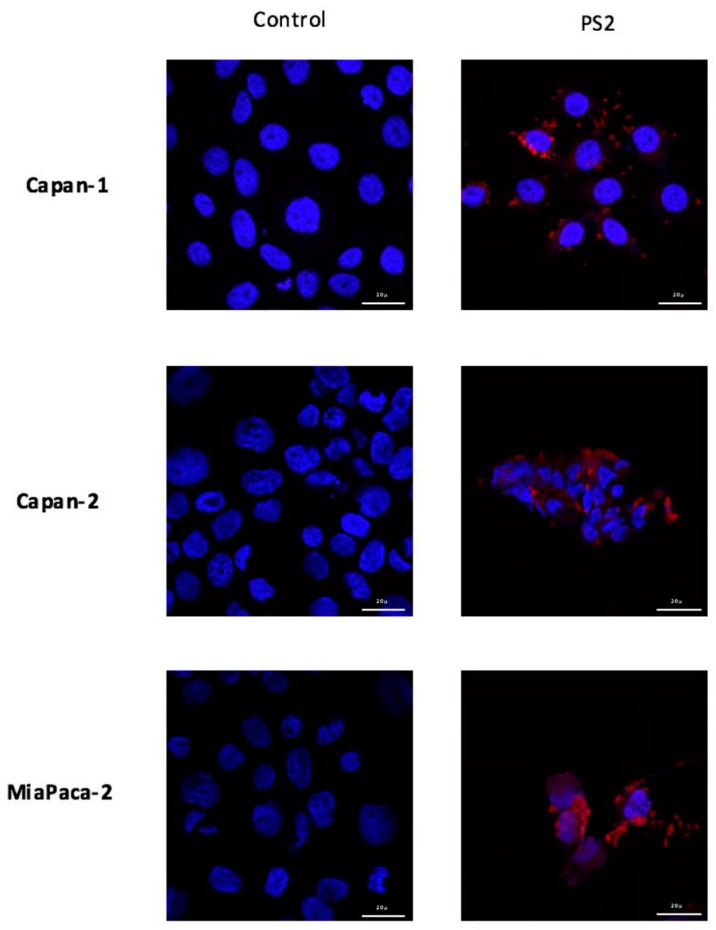

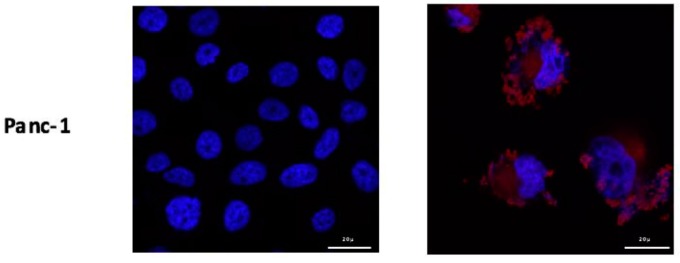
Localization of photosensitizer (PS)-2 in four PDAC cell lines. Cells are cultured with 1 µg/mL of PS2 during 24 h. Images are obtained using confocal microcopy 63× magnification.

In order to investigate the fixation of PS2 on the four human ADKP cell lines, we incubated them with 1 µg/mL of PS2 for 24 h. We can see that PS2 is mainly located at the plasma membrane level, in particular for the MiaPaCa-2 and Panc-1 cell lines but also at the intracellular level for all the cell lines. In addition, it would appear that lines Panc-1 and MiaPaCa-2 cell lines fix more PS2 than Capan-1 and Capan-2 lines (Figure 2).

### 3.3. Impact of PS2-PDT on the Viability of PDAC Cancer Cell Lines

**Figure 3:**
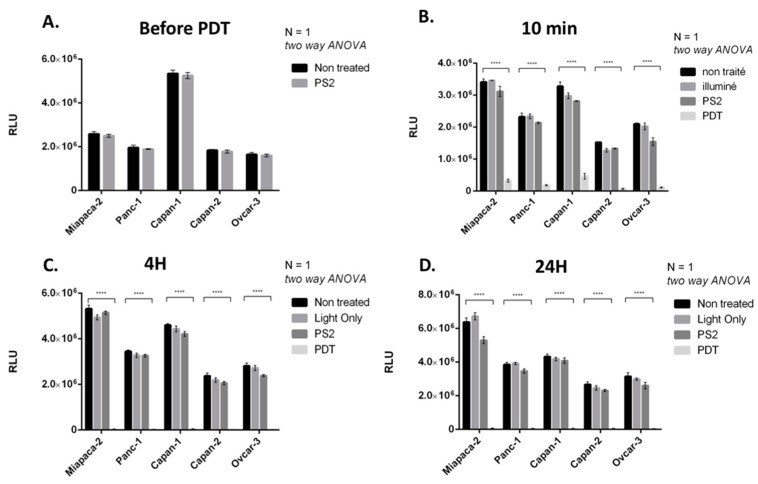
Impact of PS2-PhotoDynamic Therapy (PDT) on the four PDAC cell lines and one ovarian cancer viability. (**A**) Before illumination, (**B**) 10 min after illumination, (**C**) 4 h after illumination, and (**D**) 24 h after illumination. All quoted *p*-values are two-sided, with *p* ≤ 0.00001 (****) being considered statistically significant for the first and highly significant for the others.

To evaluate the effect of PDT on the four PDAC cell lines, we performed incubation with PS2 at 10 µg/mL during 24 h. Compared to the PS2 condition and light control conditions, the cells subjected to PDT show an important decrease in their viability (95%) starting at 10 min after illumination. The viability 4 and 24 h after PDT treatment for all lines is 0% (Figure 3A–D).

**Figure 4:**
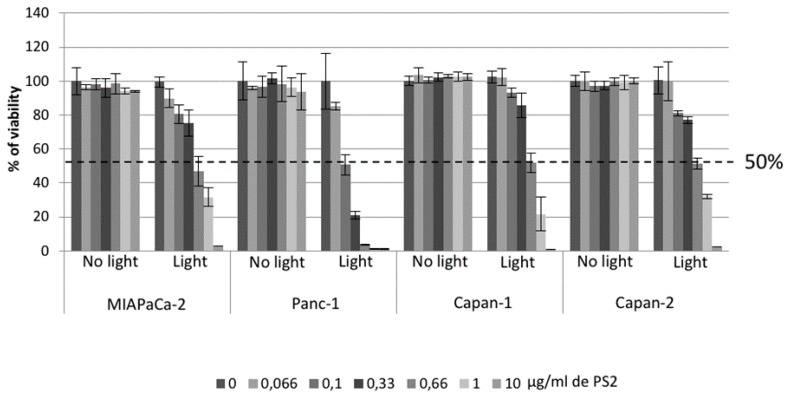
Determination of the concentration of the Lethal Dose (LD)-50. The cells were incubated at the indicated PS2 concentrations for 24 h. The viability test was carried out 24 h after PDT. The viability percentage was calculated by making a ratio of the different conditions to the untreated cells.

Then, in order to evaluate the tumour secretome we determined a concentration of PS2 that causes 50% decreased of viability (LD50). The LD50 for MiaPaCa-2, Capan-1, and Capan-2 seems to be around 0.66 and 0.166 µg/mL for Panc-1 (Figure 4).

### 3.4. Impact of PS2-PDT on the Cytokine Secretions of PDAC Cell Lines

**Figure 5:**
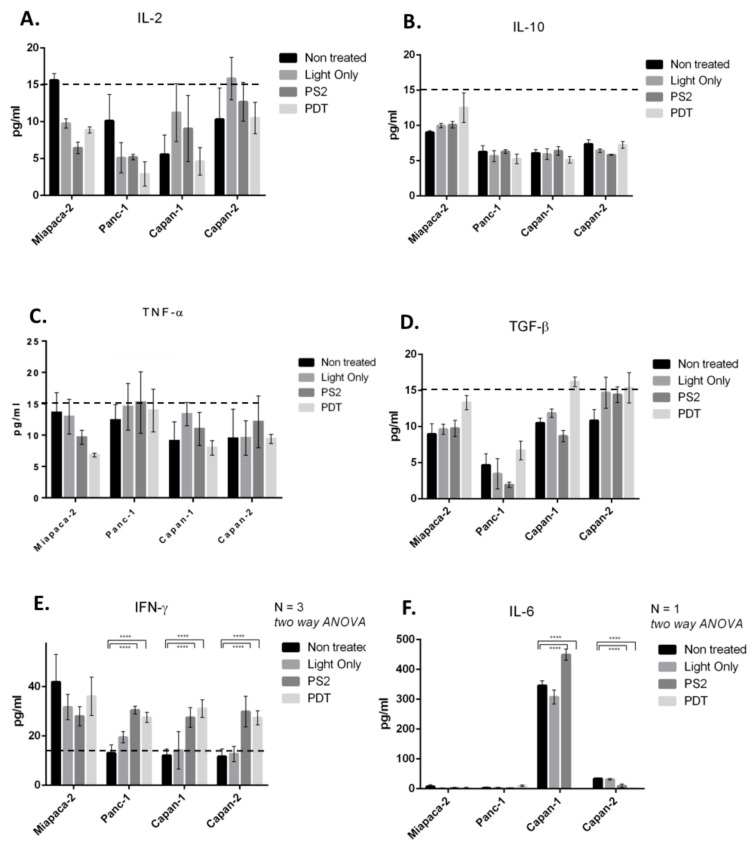
ELISA of different cytokines in the medium culture of the four cancer cell lines (**A**) Interleukine (IL)-2, (**B**) IL-10, (**C**) Tumor Necrosis Factor (TNF)-α, (**D**) Transforming Growth Factor (TGF)-β, (**E**) Interferon (IFN)-γ, and (**F**) IL-6 detection by ELISA. Dotted line represent the limit of quantification threshold. All quoted *p*-values are two-sided, with *p* ≤ 0.00001 (****) being considered statistically significant for the first and highly significant for the others.

The results show that IL-2, IL-10, TNF-α, and TGF-β production is less than 15 pg/mL. These values being lower than the detection limit of our ELISAs, it would seem that the lines produce few of these cytokines (Figure 5A–D).

Regarding IFN-γ, after PDT treatment an increase of 50% is observed with Capan-1, Capan-2, and Panc-1 cell lines when compared to the non-treated condition. Interestingly the same result is also described with PS2 condition. Regarding the MiaPaCa-2 cell line a small decrease of IFN-γ production is observed with light, PS2, and to a lesser extent in PDT condition (Figure 5E).

For IL-6 production, MiaPaCa-2 and Panc-1 cell lines show a low production of IL-6, less than 15 pg/mL. However, we can see that Capan-1 cell line produces a lot of IL-6 in standard condition (untreated) and that this production increases slightly with PS2, but becomes null after PDT. In a lesser extent the same result is also observed with Capan-2 (Figure 5F).

### 3.5. Effect of the Conditioned Medium of PDAC Cell Lines on the Proliferation of Human PBMC

In order to assess the impact of PDAC cell lines secretion on the proliferation of human PBMCs, we co-cultured PBMCs with the conditioned media of MiaPaCa-2 cell line under different conditions.

**Figure 6:**
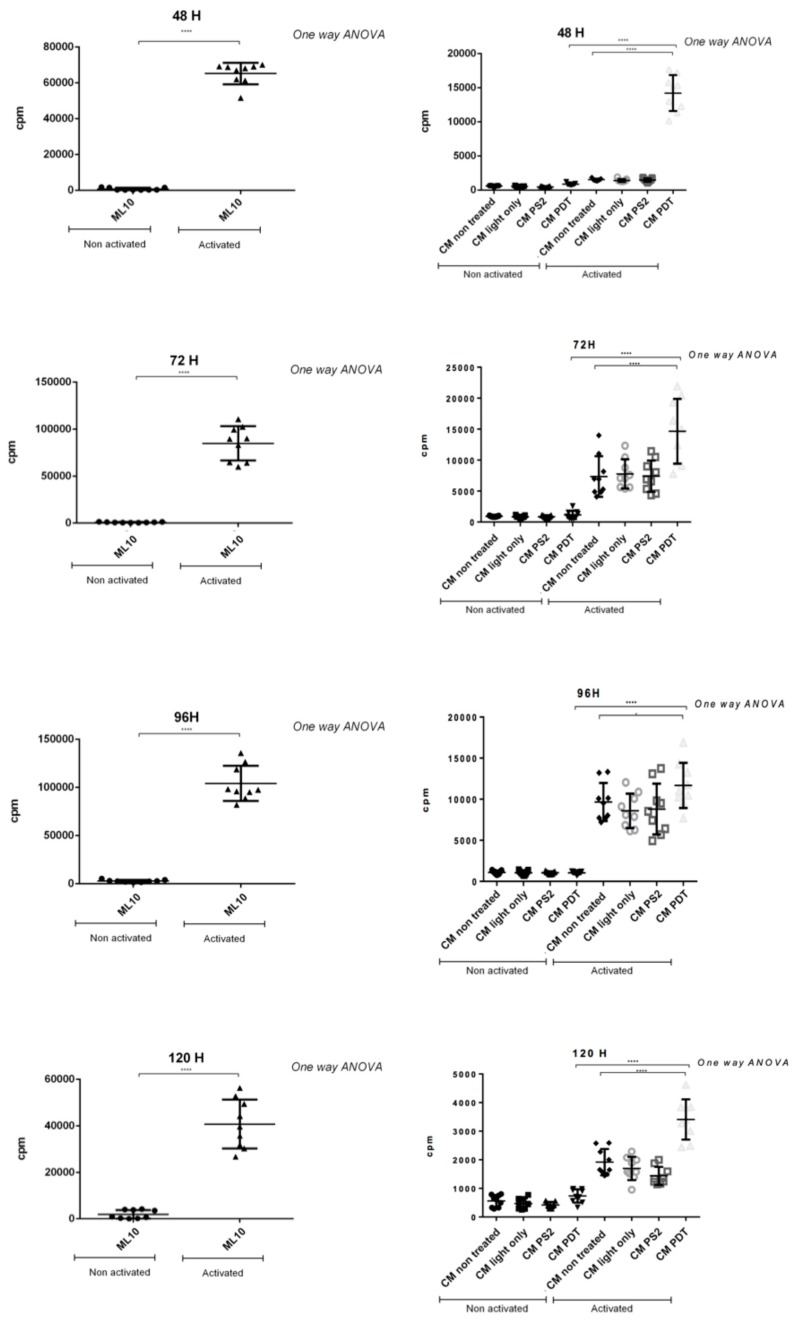
Peripheral Blood Mononuclear Cells (PBMC) proliferation tests in the presence of conditioned media from the MiaPaCa-2 lineage. The PBMCs were activated with an anti-CD3–CD28 cocktail and incubated in the presence of the conditioned media (CM) for 48, 72, 96, and 120 h. All quoted *p*-values are two-sided, with *p* ≤ 0.05 (*), *p* ≤ 0.001 (**), *p* ≤ 0.0001 (***), and *p* ≤ 0.00001 (****) being considered statistically significant for the first and highly significant for the others.

The results show, initially, that PBMCs activated with anti-CD3 and anti-CD28 are well able to proliferate. On the other hand, and as expected, significant inhibition of this proliferation is observed after contact with conditioned media of “untreated” PDAC cells (Figure 6).

Very interestingly and in comparison with the previous condition, there is a significant increase in the proliferation of these PBMCs (activated or not) when they are co-cultured with conditioned medium (MC) of cancer cells treated with “PDT” (Figure 6).

### 3.6. In Vivo Evaluation of the PDT Efficiency in the Humanized SCID Mice Model of Pancreatic Cancer

**Figure 7:**
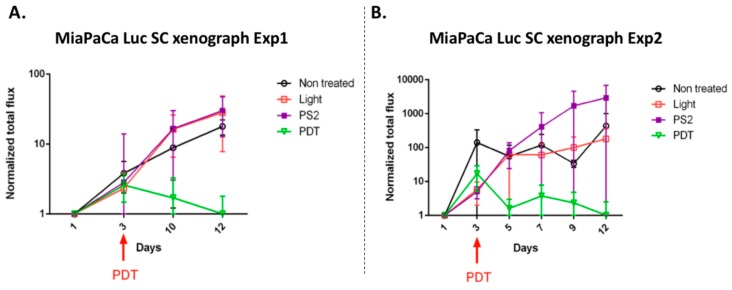
In vivo evaluation of PDT efficacy in a humanized Severe Combined ImmunoDeficiency (SCID) mice model of pancreatic cancer in one set of experiment (**A**) and for a second set of experiment (**B**). *n* = 3 per condition per experiment.

We decided to evaluate the PDT efficacy in a humanized SCID mouse model of PDAC developed in the laboratory. According to the laboratory protocol, SCID mice were xenotransplanted with the human cell line MiapaCa-2 Luc and then subjected to a different treatment. The mice received either PS2 alone, light alone, PS2 and light alone (PDT), and finally one group received nothing. Light alone or PS2 does not seem to affect tumor growth. Very interestingly, we can observe in two independent experiments that the PS2-PDT significantly limited tumor growth in this model (Figure 7A,B).

## 4. Discussion

In the present study, our goal was to evaluate the efficacy for photodynamic therapy of a new photosensitizer (PS2) specifically bound to folic acid. We evaluated the impact of PDT on four different cell lines of PDAC. We also investigated the consequences of this treatment on the activation of the human immune system. First, the analysis of the expression of the FOLR1 showed that all the cell lines express it. According to the previous studies, FOLR1 might be a promising target for novel therapeutic or diagnostic strategies [13]. We compared the folate expression of the different cancer cell lines with the OVCAR3 cell line, known to have an important expression of folate receptor. Moreover, the flow cytometry analysis of the protein expression of FOLR1 supported our qPCR.

Furthermore, in order to visualize the binding of the new PS on the cells, we conducted immunofluorescence analysis of the PS2 cultured with the different cells. Our preliminary results suggest that the PS binds to all different cell lines with a slight preference for MiaPaCa-2 and Panc-1 cell lines. Moreover, we can see that PS2 preferentially binds to the membrane of the cells. However, we can detect some intracellular labelling suggesting an internalization of the PS2. In fact, this intracellular presence of the folate receptor has been previously described [17]. This internalization is due to the endocytosis mechanisms responsible of the folate absorption [18]. Thus, the presence of folate receptors on the different tumor cell lines suggests that they would potentially be sensitive to the new patent-pending photosensitizer.

In order to confirm the effect of PDT using the photosensitizer on ovarian tumor cells, we first evaluated the viability of our different cancer cell lines after PDT. Four conditions were tested: Untreated, cell culture with PS (PS2), cells subjected to illumination (Light only), and cells subjected to illumination in the presence of PS2 (PDT). We noticed that MiapaCa-2, Panc-1, Capan-1, and Capan-2 subjected to PDT presented a significant decrease in their cell viability over time starting at 10 min post-illumination. On the other hand, there are no notable changes in the viability of untreated, PS2-only, or illumination-only tumor cells. Several other photosensitizers have been studied for their potential effect on pancreatic cancer treatment [19,20,21]. The most important difference between our PS and the other photosensitizer lies in its capacity to localize in the cancer cells highly expressing the folate receptor. In addition, in order to determine the LD50, we performed PDT using different PS2 concentrations. Our results suggested that the LD50 for MiaPaCa-2, Capan-1, and Capan-2 appears to be 0.66 µg/mL whereas; the LD50 for Panc-1 appears to be 0.1 µg/mL.

These concentrations will then be used to study the cytokines expression upon PDT. Our results do not show any increase in the production of IL-2. On the other hand, our results show that PDT does not induce the production of immunosuppressive cytokines IL-10 and TGF-β. In fact, the unfavorable role of these cytokines in cancer have been previously described [22]. In fact, IL-10 acts as a mediator of the anti-inflammatory response and as an inhibitor of macrophages and dendritic cells (DCs) [23], and TGF-β is known to act as a pro-tumorigenic by induction of an epithelial-mesenchymal transition (EMT) in pancreatic adenocarcinoma [24]. Concerning the production of IFN-γ, another anti-tumor effector cytokine, our results suggest that for Capan-1, Capan-2, and Panc-1 lines, the production of IFN-γ increase when the cells are subjected to PDT or when cultured with PS2 only. Very interestingly, we have shown that PDT seems to neutralize the production of IL-6 from the Capan-1 cell line. However, it is known that PDAC is an inflammatory cancer and patients show an increase in a certain number of cytokines, including IL-6. The consequence is an inflammation, mediated mainly by IL-6, locally generating an immunosuppressive microenvironment responsible for the escape of tumor cells and their dissemination [25]. PDT using the new PS seems to counterbalance this effect. In addition, it has been previously described that in PDAC IL-6 facilitates migration, invasion by tumor cells, and eventually epithelial-mesenchymal transition. In addition, inhibition of IL-6 in several murine models of PDAC has limited tumor growth [26]. In the context of an inflammatory cancer such as PDAC, the neutralization of the inflammation would be a major asset for the establishment of an effective immune response.

Furthermore, to evaluate the impact of PDAC cell secretome after PDT on immunity, PBMCs have been co-cultured with the conditioned media of the different conditions of cell culture. Our results show that when the PBMCs are activated an inhibition of their proliferation is observed after culture with conditioned media of untreated cells. In a very interesting way, we observe a significant increase in their proliferation when cultured with conditioned media of cancer cells subjected to PDT. Our result suggests an immunostimulant effect induced by cells treated with PDT.

Finally, in order to evaluate in vivo the impact of the new PS2 we analyze the tumor growth in a humanized SCID mice model of pancreatic cancer. Four in vivo conditions were tested: Untreated, mice (nontreated), mice with PS (PS2), mice subjected to illumination (Light only), and mice subjected to illumination in the presence of PS (PDT). We noticed that the mice subjected to PDT presented a strong decrease in the growth of the tumor over time after illumination. On the other hand, there are no notable changes in the growth of the tumors in the nontreated, light only, and PS-only groups.

In conclusion, the development of a new generation of PS targeting PDAC cells via the folate receptor-α, allows us to consider the treatment of these cells by PDT. Our investigations had not only suggested that PDT by PS2 is an effective therapy in the treatment of PDAC but also that it activates the immune system and be a huge adjuvant for anti-cancer vaccination. Thus, this new study will provide new treatment options for patients in a therapeutic impasse and will provide a new arsenal in the fight against PDAC.

## Figures and Tables

**Table 1:** List of the primers used for QPCR.

FOLR1	5′-AGGTGCCATCTCTCCACAGT	5′-GAGGACAAGTTGCATGAGCA
18S	5′-TCAAGAACGAAAGTCGGAGG	5′-GGACATCTAAGGGCATCACA
GAPDH	5′-GCCAAGGTCATCCATGACAACTTTGG	5′-GCCTGCTTCACCACCTTCTTGATGTC
HPRT	5′-CCCTGGCGTCGTGATTAG	5′-ATGGCCTCCCATCTCCTT

## Supplementary Materials

The following are available online at https://www.mdpi.com/2077-0383/9/1/192/s1, Figure S1: Structural formula of the compound Pyro-PEG-FA (PS2).
