# Supplementary figures and images for: An Efficient Photodynamic Therapy Treatment for Human Pancreatic Adenocarcinoma
Source: J Clin Med. 2020 Jan 10;9(1):192. doi: 10.3390/jcm9010192 (PMC7019594; doi:10.3390/jcm9010192)

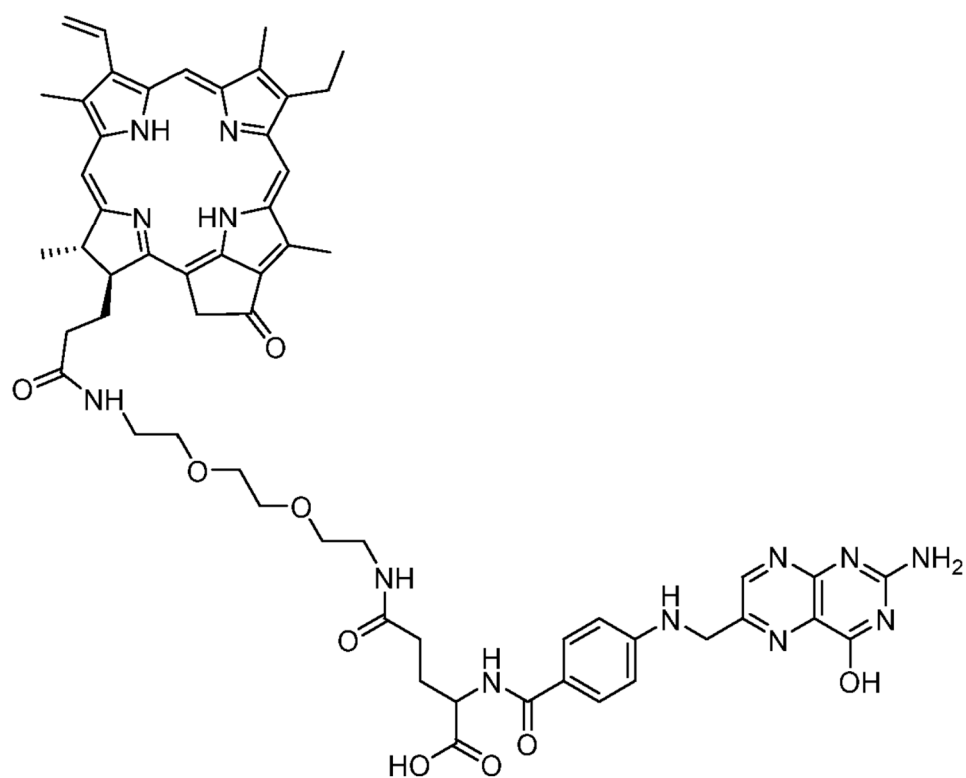

**Figure S1.** Structural formula of the compound Pyro-PEG-FA (PS2).

Supplement: Supplementary file 1 [file jcm-09-00192-s001.pdf]
